# Selective Targeting of Serotonin 5-HT1a and 5-HT3 Receptors Attenuates Acute and Long-Term Hypersensitivity Associated With Neonatal Procedural Pain

**DOI:** 10.3389/fpain.2022.872587

**Published:** 2022-04-27

**Authors:** Anne R. de Kort, Elbert A. Joosten, Jacob Patijn, Dick Tibboel, Nynke J. van den Hoogen

**Affiliations:** ^1^Department of Anesthesiology and Pain Management, Maastricht University Medical Centre+, Maastricht, Netherlands; ^2^Department of Translational Neuroscience, School of Mental Health and Neuroscience, Maastricht University, Maastricht, Netherlands; ^3^Intensive Care and Department of Pediatric Surgery, Erasmus MC-Sophia Children's Hospital, Rotterdam, Netherlands; ^4^Department of Comparative Biology and Experimental Medicine, Hotchkiss Brain Institute, University of Calgary, Calgary, AB, Canada

**Keywords:** neonatal pain, procedural pain, treatment, serotonin, ondansetron, buspirone

## Abstract

Neonatal painful procedures causes acute pain and trigger long-term changes in nociceptive processing and anxiety behavior, highlighting the need for adequate analgesia during this critical time. Spinal serotonergic receptors 5-HT1a and 5-HT3 play an important role in modulating incoming nociceptive signals in neonates. The current study aims to attenuate acute and long-term hypersensitivity associated with neonatal procedural pain using ondansetron (a 5-HT3 antagonist) and buspirone (a 5-HT1a agonist) in a well-established rat model of repetitive needle pricking. Sprague-Dawley rat pups of both sexes received ondansetron (3 mg/kg), buspirone (3 mg/kg) or saline prior to repetitive needle pricks into the left hind-paw from postnatal day 0–7. Control animals received tactile stimulation or were left undisturbed. Acute, long-term, and post-operative mechanical sensitivity as well as adult anxiety were assessed. Neonatal 5-HT1a receptor agonism completely reverses acute hypersensitivity from P0-7. The increased duration of postoperative hypersensitivity after re-injury in adulthood is abolished by 5-HT3 receptor antagonism during neonatal repetitive needle pricking, without affecting baseline sensitivity. Moreover, 5-HT1a and 5-HT3 receptor modulation decreases adult state anxiety. Altogether, our data suggests that targeted pharmacological treatment based on the modulation of spinal serotonergic network via the 5-HT1a and 5-HT3 receptors in neonates may be of use in treatment of neonatal procedural pain and its long-term consequences. This may result in a new mechanism-based therapeutic venue in treatment of procedural pain in human neonates.

## Introduction

Newborns admitted to the neonatal intensive care unit (NICU) can be exposed to repetitive nociceptive input during a vulnerable time frame for the central nervous system, as part of their care and treatment. The neonatal period is unique in how it processes nociceptive stimuli, and the nociceptive network undergoes developmental changes in early life (1–3). Due to its activity-dependent maturation, the nociceptive network develops differently when exposed to excessive noxious input in early life (4–7). Both clinical and fundamental studies have provided evidence for neonatal painful stimulation induced alterations in the developing nociceptive network, resulting in subsequent long-term alterations in pain sensitivity (3, 8–11) and exacerbated sensitivity after re-injury in adulthood (12–17). Neonatal procedural pain induced effects are not limited to the nociceptive network but also affect anxiety behavior amongst others (18–23), suggesting a common neurocircuitry that regulates both pain and anxiety behavior is involved.

Serotonin (5-hydroxytryptamine, 5-HT) is a key modulator of anxiety behavior and perception of pain, and its ascending and descending fibers are distributed throughout the brain and spinal cord (24). Virtually all serotonergic innervation of the spinal cord originates from supraspinal sources in the brainstem, specifically the nucleus raphe magnus (NRM), which provides serotonergic input to the spinal dorsal horn (25). Based on the receptors involved, the net effect of serotonergic descending modulation might be either inhibitory or facilitatory (26). In the healthy adult rodent, serotonin is inhibiting spinal nociceptive neurotransmission (26).

Descending serotonergic modulation of the spinal nociceptive network, originating in the rostral ventromedial medulla (RVM), is immature in early life and undergoes developmental changes in anatomy and functionality. Importantly, the functionality switches from facilitation to inhibition of nociceptive signaling in early life (26, 27). Since treatment of neonatal pain is still a clinical challenge, analgesic therapy that considers the neurodevelopmental phase may result in more targeted treatment of neonatal procedural pain (27). In this study, we focus on two serotonin receptors: the inhibitory 5-HT1a and the excitatory 5-HT3. The serotonin receptors 5-HT1a is a Gi-coupled, transmembrane receptor expressed in the spinal dorsal horn, mainly on nociceptive afferents and inhibitory interneurons. When activated, the 5-HT1a receptor leads to inhibition of nociceptive signaling through a reduced glutamate release from primary afferent terminals (28). The 5-HT3 is a ligand-gated ion channel, expressed on nociceptive afferents, projection neurons, and both inhibitory and excitatory interneurons. Activation of this receptor leads to antinociception through activation of inhibitory interneurons in the spinal cord, who in turn release GABA and reduce transmission of nociceptive signals. However, 5-HT3 is also been implied in facilitation of nociceptive stimuli, especially in the neonatal period, through its expression on wide dynamic range (WDR) secondary nociceptive afferents (25).

During the neonatal period, facilitation of nociceptive signaling is mediated via the 5-HT3 receptors (R) in the dorsal horn (28–30) whereas the 5-HT1aR plays a role in inhibition of nociception (25, 28, 29, 31, 32). Therefore, activation of the 5-HT1aR or inactivation of the 5-HT3R in neonates may provide neurodevelopmental relevant analgesia, and could restore injury-evoked disruptions in the normal balance of excitation and inhibition in the spinal dorsal horn (17, 33–35). In addition, early postnatal signaling of the 5-HT1aR is required for normal development of anxiety-related brain areas and behavior (36, 37). Antagonizing the 5-HT3R in adulthood shows promising anxiolytic effects (38). Targeting the 5-HT3R and 5-HT1aR in newborns may therefore attenuate both nociception and anxiety related long-term consequences of neonatal painful stimuli, while providing adequate acute analgesia.

The aim of this study is to prevent the acute and long-term consequences of repetitive neonatal procedural pain by pharmacological targeting the serotonin 5-HT1aR or 5-HT3R in neonates. Here, we used buspirone, a full agonist of the 5-HT1aR to produce inhibition (39), or ondansetron, a selective 5-HT3R antagonist to prevent facilitation (40), in a well-established rat model of neonatal repetitive needle pricking. The effects of these interventions are studied in the context of acute, long-term and post-operative pain (or mechanical sensitivity) as well as adult anxiety.

## Materials and Methods

### Animals

All experiments and procedures were performed in line with the European Directive for Protection of Vertebrate Animal Used for Experimental and Other Scientific Purposes (2010/63/EU), and were ethically approved by the Committee for Experiments on Animals, Maastricht University, Maastricht, the Netherlands (DEC protocol 2017-017). Adult Sprague-Dawley rats were purchased from Charles River Laboratory (Sulzfeld, Germany), and were mated at Maastricht University. Animals were housed under reversed day-night conditions (12:12 h, lights on 7 p.m.– 7 a.m.), in temperature (21 ± 1°C) and humidity (55 ± 15%) controlled rooms with food and water available ad libitum. Since the circadian rhythm of animals seems capable of affecting pain sensitivity, especially mechanical sensitivity [reviewed in (41)], all behavioral experiments were performed in the active, and thus dark phase. Pups were collected and sexed at birth [postnatal (P) day 0], and litters were culled to a maximum of 10 pups to ensure equal caretaking by the dam. Conditions were distributed equally over litters and sexes (Table 1). Pups were weaned at P21 and randomly housed in same-sex groups of two or three in individually ventilated cages for the remainder of the study. The animal order of behavioral testing was randomized, and researchers were blinded to treatment groups throughout the entire experimental protocol. Behavioral testing and surgeries were performed by the same researcher.

**Table 1:** Distribution of sex and condition in experimental litters.

	**UD**		**TC**		**NP**		**NP + BUS**		**NP + OND**	
**Litter**	***F***	***M***	***F***	***M***	***F***	***M***	***F***	***M***	***F***	***M***
1			1	1	1	1	3	3		
2			1	1	1	1	3	3		
3			1	1	1	1			3	3
4			1	1	1	1			3	3
5	5	5								
6			2	2	2	2				2

*F, female; M, male; NP, needle prick animals; NP + BUS, needle prick animals receiving buspirone; NP + OND, needle prick animals receiving ondansetron; TC, tactile controls; UD, undisturbed control*.

### Neonatal Procedures

On the day of birth (P0) newborn pups were randomized, using a computer-generated randomization list, to one of four conditions; 1. repetitive needle pricking (needle pricking, NP; *n* = 12), 2. NP with buspirone treatment (NP+BUS; 3 mg/kg; *n* = 12), 3. NP with ondansetron treatment (NP+OND; 3 mg/kg; *n* = 13) or 4. repetitive tactile stimulation (TC; *n* = 12). A separate litter was left completely undisturbed (UC; *n* = 10). Rat pups (conditions 1–3) were noxiously stimulated with repetitive needle pricks (NP) four times a day at 09:00 a.m., 10:00 a.m., 11:00 a.m. and 12:00 p.m. from P0 to P7 as previously described (13, 14). Each noxious stimulation consisted of a single 2 mm deep needle prick with a 25G needle in the mid-plantar surface of the left hind paw. Age-matched littermates used for the tactile stimulation (TC) (condition 4) received four tactile stimulations by stroking the left hind haw with a cotton tip swab at similar intervals. Drug doses (50 μl) were administered subcutaneously (sc.) 10 min before the first and third NP to cover the entire period of repetitive needle pricking. TC and NP animals received sc. saline to control for injection procedures and handling. Mechanical sensitivity was assessed before (baseline, BL) and 1, 3, and 5 h after the last needle prick or tactile stimulation from P0 to P7, using dorsal application of calibrated Von Frey filaments [bending force 0.407, 0.692, 1.202, 2.041, 3.63, (from P4 onwards) and 5.495 (from P6 onwards)].

### Drugs

**Figure 1:**
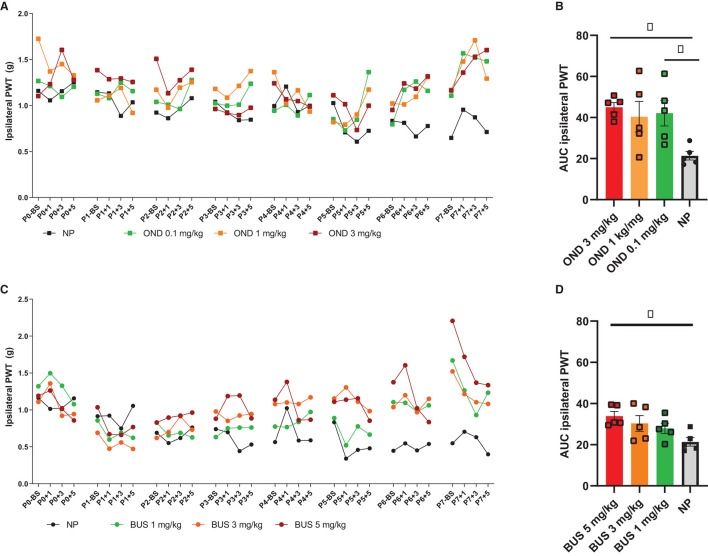
Dose-response pilots of ondansetron (0.1, 1, and 3 mg/kg) and buspirone (1, 3, and 5 mg/kg). **(A)** PWTs increase over time, but not equally for all conditions [F (93, 636) = 4.841; *p* < 0.01]. Repetitive needle pricking (NP; *n* = 12) results in a decreased paw-withdrawal threshold (PWT) over time, that was significantly different from OND 3 mg/kg from P5 onwards. **(B)** The area under the curve (AUC) over the entire neonatal period is significantly different between neonatal conditions for the ipsilateral paw-withdrawal thresholds (PWT) [F (3, 16) = 4.432; *p* = 0.0189]. AUC was significantly increased in NP animals after administration of 0.3 mg/kg (*p* = 0.0329) and OND 3 mg/kg (*p* = 0.0143), as compared to NP animals receiving saline. **(C)** PWTs increase over time, but not equally for all groups [F (93,496) = 2.600; *p* < 0.01]. Repetitive needle pricking (NP) results in decreased PWTs over time, that was significant different from BUS 1 mg/kg from P5 onwards, from BUS 3 mg/kg from P4 onwards and BUS 5 mg/kg from P3 onwards. **(D)** The AUC over the entire neonatal period is significantly different between conditions [F (3, 16) = 3.745; *p* = 0.0327]. NP animals receiving BUS 5 mg/kg showed significantly increased AUC as compared to NP animals receiving saline (*p* = 0.0151). BS, baseline measurement; NP, needle pricking animals; BUS, buspirone; OND, ondansetron; P (0–7), postnatal day 0–7; +1, +3, +5, measurement 1, 3 and 5 h after needle prick or tactile stimulation. Data is presented as mean ± SEM, NP vs. NP+BUS. ^*^P < 0.05.

For 5-HT3R antagonism, the selective 5-HT3R antagonist Ondansetron (OND; ondansetron hydrochloride dihydrate, Sigma-Aldrich, O3639) was dissolved in sterile saline in a concentration of 2 μg/μl. For 5-HT1aR agonism, the selective agonist Buspirone (BUS, buspirone hydrochloride; Sigma-Aldrich, B7148) was dissolved in sterile saline to a concentration of 10 μg/μl. Pilot studies testing 1, 3 and 5 mg/kg BUS and 0.3, 1 and 3 mg/kg OND showed that 3 mg/kg BUS and 3 mg/kg OND were most promising in preventing acute hypersensitivity associated with neonatal repetitive needle pricking (see Figure 1), without adverse side effects (including body weight changes or mortality). Based on pup's body weight per postnatal day, 50 μl solution containing OND (3 mg/kg) or BUS (3 mg/kg) was injected sc. twice daily (at 9 a.m. before the 1st needle prick and 11 a.m. before the 3 rd needle prick). TC and NP animals received an equivalent volume of saline.

### Developmental Assessment

To assess the effect of neonatal 5-HT1aR agonism and 5-HT3R antagonism on general postnatal development, physical developmental milestones such as pinna detachment (unfolding of the external ear), incisor eruption (of lower and upper teeth), fur development, complete opening of both ears and complete opening of both eyes were expressed as the number of postnatal days required for the appearance of these milestones (42). These milestones were assessed every 24 h at 1 p.m. (after VF testing to reduce separation), until all pups of a given litter developed all milestones. The surface righting reflex was tested from P1 to P5, to assess reflex development and motor function. Pups were placed in a supine position on a flat surface and then released. The righting reflex is defined as the number of seconds required for a newborn pup to turn over on all four limbs, and was considered completed if the pup did so within 30 s (43).

### Adult Re-incision Model

To assess acute post-operative hypersensitivity, a plantar hind-paw incision surgery (or Brennan model for postoperative pain) was performed on all animals (44). Briefly, under isoflurane anesthesia (4–5% induction, 2% maintenance), a 1 cm longitudinal incision through the skin and fascia of the ipsilateral paw was made, followed by the elevation and incision (1–2 mm at midline) of the plantaris muscle. The skin was closed using two mattress sutures (5.0 Ethicon Ethylon, Somerville, New Jersey).

### Von Frey Test for Mechanical Sensitivity

Mechanical sensitivity was assessed by determining the paw withdrawal thresholds (PWT) of the hind paws to calibrated Von Frey filaments. Briefly, animals were placed in a transparent box resting on an elevated mesh floor. After a 15-min acclimation period, a series of von Frey filaments (bending forces 1.202, 2.041, 3.63, 5.495, 8.511, 15.136, and 28.84 g; Stoelting, USA) were applied to the plantar surface of the hind paw for 5 s using the up-down method (45). Mechanical sensitivity was assessed weekly from 3 to 8 weeks of age, and every other day after adult re-injury (at 1, 3, 5, and 7 days post-incision). The animal order of behavioral testing was randomized, and researchers were blinded to treatment groups during behavioral testing and surgery throughout the entire experimental protocol.

### Open Field Test

The open field test was used to evaluate locomotor activity and anxiety-related behavior in adulthood (at 8 weeks of age) (23). Briefly, individual animals were placed into the center of a plexiglass square arena (100 × 100 × 40cm), and allowed to explore the environment for 20 min. The arena was thoroughly cleaned with 70% ethanol in between sessions. A camera mounted directly above the maze recorded the behavior of the animal, analyzed by the image tracking system (Noldus Ethovision XT software, Noldus Information Technology, the Netherlands). Variables included time spend in the center (%) and center crossings (number of entries from and to the center) for the first 5 min, and the total distance traveled (locomotor path in whole arena in cm) for a 20-min duration.

### Elevated Zero Maze

The Elevated Zero Maze (EZM) was used to assess anxiety-related behavior (23). Briefly: a black annular maze (100 cm diameter, 10 cm path width) was placed 70 cm above floor level, with four equal quadrants (two opposite quadrants are “closed” by black Perspex walls, two remaining quadrants are “open”). Animals were placed in an open arm facing one of the closed arms and allowed to explore the maze for 5 min. The arena was thoroughly cleaned with 70% ethanol between sessions. An infrared camera mounted directly above the maze recorded the behavior of the animal, analyzed by the image tracking system (Noldus Ethovision XT software, Noldus Information Technology, the Netherlands). Variables included time spend in open and closed arms, latency to enter each arm, and open arm entries. The time spend in the open arms as a percentage of total (corrected) trial time (= total trial duration—latency to enter first closed arm) was classified as anxiety-like behavior.

### Statistical Analysis

All data are presented as mean ± SEM and plotted using Graphpad Prism 9 (GraphPad Software, San Diego, USA). A *P*-value <0.05 was considered statistically significant. Mechanical force resulting in a 50% withdrawal frequency was assigned as the PWT and was calculated using a sigmoid curve fitting in Graphpad Prism 9. For the neonatal period, an area under the curve (AUC) analysis was performed. The AUC was calculated based on the PWTs for each group over the entire neonatal period (P 0–7) and statistically compared using a one-way ANOVA with Bonferroni *post-hoc* correction. Differences in PWT in the first neonatal week, mechanical sensitivity throughout development, and post-operative mechanical sensitivity were analyzed using a repeated measures analysis of variance (ANOVA) with Bonferroni *post-hoc* correction. Both within- and between-group analysis were run. A non-parametric Kruskal-Wallis tests was used to compare differences in developmental milestones between conditions. A two-way ANOVA was performed to compare the effects of condition and sex on anxiety-like behavioral measurements. For all analysis, the assumptions for statistical analysis were checked and met. For all behavioral analysis but the anxiety-like behavioral testing, the sex effect was tested and not significant, therefore data of both sexes was pooled to increase statistical power.

## Results

### Early Postnatal Developmental Milestones Are Unaffected by Neonatal 5-HT1aR Agonism or 5-HT3R Antagonism

**Figure 2:**
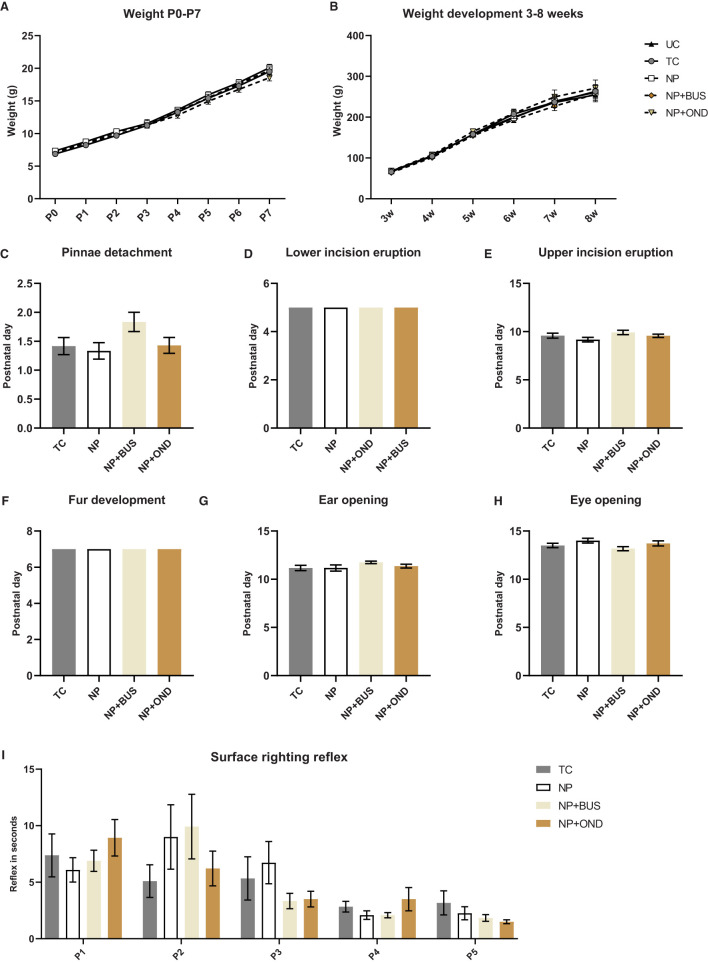
Developmental milestones and weightduring the first postnatal week and during development from 3–8 week of age. **(A)** Weight during the first postnatal week significantly increases over time [F (2.541, 116.9) = 2628.0; *p* < 0.01], but is not significantly different between conditions [F (3, 46) = 0.6406; *p* = 0.5928]. **(B)** Weight increases significantly with time during development [F (1.105, 59.65) = 579.6; *p* < 0.01] but does not significantly differ between conditions [F (4, 54) = 0.3339; *P* = 0.8540]. Developmental milestones pinnea detachment [**(C)**; H (3) = 5.550; *p* = 0.1357]; lower [**(D)**; same time point in all groups, *p* > 0.999] and upper [**(E)**; H (3) = 4.184; *p* = 0.2423] incision eruption; fur development [**(F)**; same time point in all groups, *p* > 0.999]; ear opening [**(G)**; H (3) = 2.992; *p* = 0.3928]; eye opening [**(H)**; H (3) = 5.163; *p* = 0.1602] were not different between neonatal conditions. **(I)** The surface righting reflex developed in a similar manner in all groups [F (3, 46) = 0.08918; *p* = 0.9656], while postnatal day had a significant effect [F (2.362,101.6) = 15.27; *p* < 0.001]. NP, needle prick animals; NP+BUS, needle prick animals receiving buspirone; NP+OND, needle prick animals receiving ondansetron; PWT, paw withdrawal threshold; TC, tactile control animals; UD, undisturbed control animals. Data is presented as mean ± SEM, *P < 0.05 **P < 0.01.

Pinnae detachment, lower and upper incisor eruption, fur development, ear opening and eye opening are unaffected by neonatal condition (*p* > 0.05; Figures 2C–H). In addition, no significant differences between conditions in their surface righting reflex [F (3, 46) = 0.0232; *p* = 0.9951; Figure 2I] or weight [F (3, 46) = 0.6406; *p* = 0.5928; Figures 2A,B] during the first postnatal week are observed.

### Neonatal 5-HT1aR Agonism, but Not 5-HT3R Antagonism, Prevents Acute Mechanical Hypersensitivity After Repetitive Neonatal Needle Pricking

**Figure 3:**
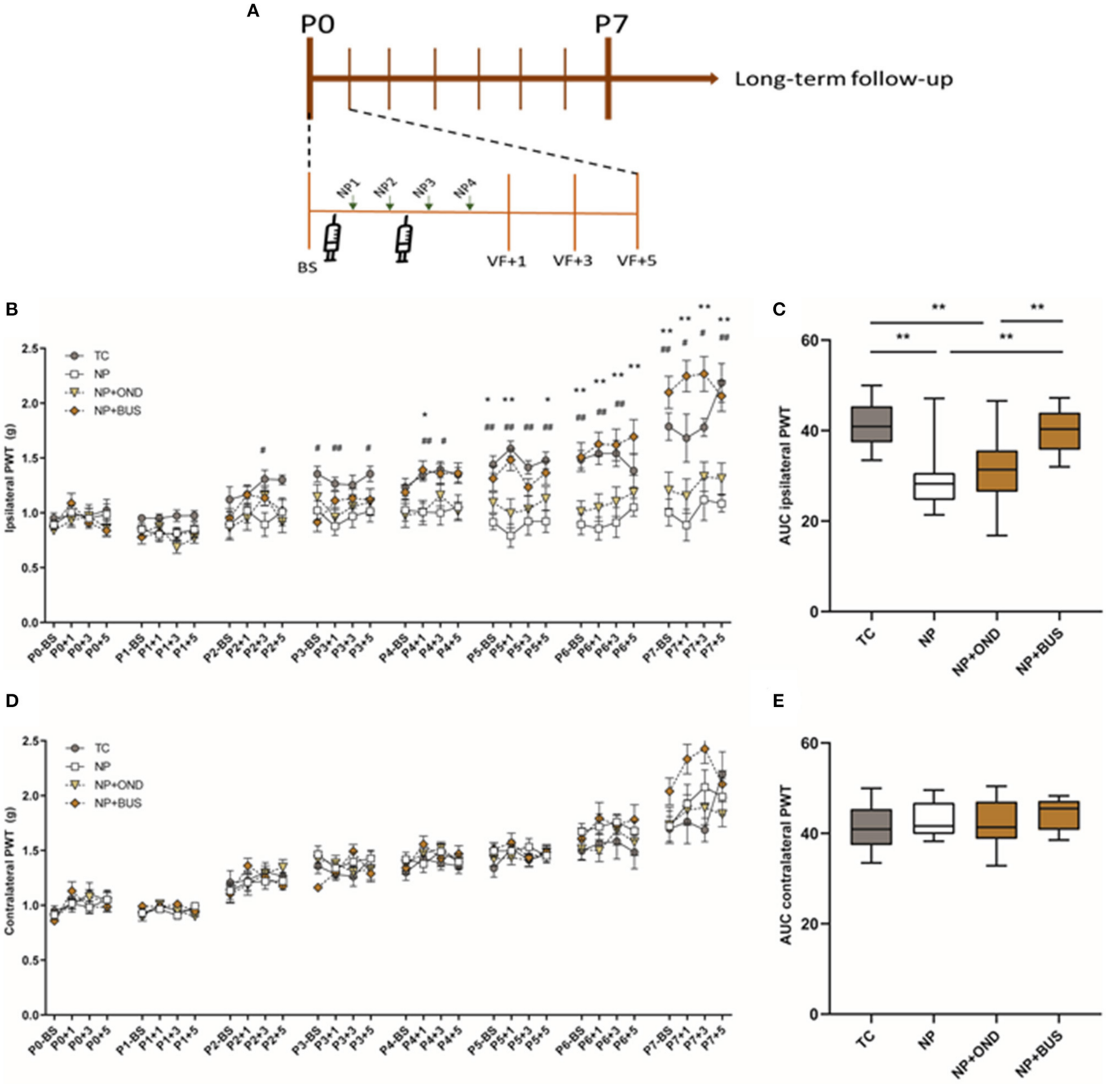
**(A)** Graphical representation of timeline of experiment. Mechanical sensitivity to von Frey filaments during first neonatal week. **(B)** Repetitive needle pricking (NP; *n* = 12) results in a decreased paw-withdrawal threshold (PWT) in the ipsilateral paw as compared to repetitive tactile stimulation (TC; *n* = 12) from postnatal day (P) 2 onwards, indicating the development of acute mechanical hypersensitivity in NP pups. Neonatal 5-HT1aR agonism (NP + BUS; *n* = 12), but not 5-HT3R antagonism (NP + OND; *n* = 14), is able to reverse the acute mechanical hypersensitivity after repetitive needle pricking [interaction effect: F (93, 1426) = 4.798; *p* < 0.001]. **(C)** The area under the curve (AUC) over the entire neonatal period is significantly different between neonatal conditions for the ipsilateral paw-withdrawal thresholds (PWT) [F (3, 46) = 10.79; *p* < 0.01]. NP as well as NP+OND animals show a decreased AUC as compared to TC animals, whereas NP+BUS reverses the decreased AUC back to TC control values. **(D)** Mechanical sensitivity of the contralateral paw increases over time all groups, but was not affected by neonatal condition [F (3, 46) = 0.9085; *p* = 0.444]. **(E)** The area under the curve analysis (AUC) over the entire neonatal period does not show significant differences in contralateral PWTs between conditions [F (3, 46) = 0.9818; *p* = 0.4096]. BS, baseline measurement; NP, needle pricking animals; NP+BUS, needle prick animals receiving buspirone; NP+OND, needle prick animals receiving ondansetron; PWT, paw withdrawal threshold; P (0–7), postnatal day 0–7; +1, +3, +5, measurement 1, 3, and 5 hours after needle prick or tactile stimulation; TC, tactile controls. Data is presented as mean ± SEM, NP vs. NP+BUS. *P < 0.05, ^**^P < 0.01, NP vs. TC, ^#^*P* < 0.05, ^##^*P* < 0.01.

During the first postnatal week ipsilateral mechanical sensitivity increases over time, but not equally for all conditions [interaction effect: F (93, 1426) = 4.798; *p* < 0.001]. *Post hoc* analysis revealed that repetitive NP results in significantly lower PWTs as compared to repetitive tactile stimulation from postnatal day 2 onwards (Figure 3A). Neonatal 5-HT1aR agonism using buspirone reverses the drop in PWTs after repetitive NP in NP+BUS animals from P4 onwards, whereas the drop in PWTs is still present after neonatal 5-HT3R antagonism using ondansetron (NP+OND; Figure 3A). The AUC analysis over the entire neonatal period reveals a significant decreased ipsilateral AUC in NP animals and NP+OND, as compared to both TC and NP+BUS animals [effect of condition: F (3, 46) = 10.79; *p* < 0.01; Figure 3B]. Contralateral mechanical sensitivity increases over time [effect of time: F (10.02, 460.7) = 62.68; *p* < 0.01] but is not significantly different between conditions [effect of condition: F (3, 46) = 0.9085; *p* = 0.444; Figure 3C]. Similarly, contralateral AUC are not significantly different between conditions [effect of condition: F (3, 46) = 0.9818; *p* = 0.4096; Figure 3D].

### Neonatal 5-HT1aR Agonism, but Not 5-HT3R Antagonism, Affects Mechanical Sensitivity During Development From Weaning to Adulthood

**Figure 4:**
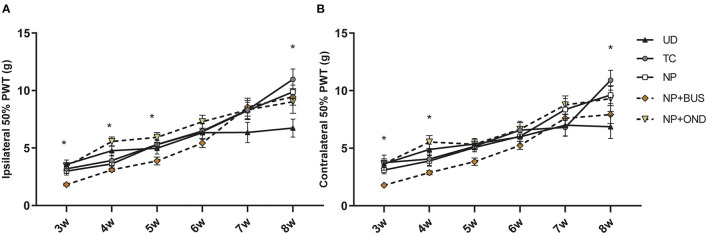
Mechanical sensitivity to von Frey filaments throughout development. **(A)** Ipsilateral PWTs increase over time for all groups [F (3.048, 164.6) = 105.2, *p* < 0.01], but show a significant time*condition interaction [F (20,270) = 2.865, *p* < 0.01]. Posthoc analysis revealed a lower PWTs for the NP+BUS animals as compared to NP (at 3 and 4 weeks), NP+OND (at 3, 4, and 5 weeks), TC (at 3 weeks) and UD animals (at 3 and 4 weeks). NP and TC animals show higher PWTs at 8 weeks as compared to UD animals. **(B)** Contralateral PWTs increase over time for all groups [F (23.319, 179.2) = 81.69; *p* < 0.01], but show a significant time*condition effect [F (20, 270) = 2.104, *p* < 0.01]. Lower PWTs for the NP+BUS animals as compared to NP (at 3 weeks), NP+OND (at 3 and 4 weeks) and UD (at 3 and 4 weeks) are noted. Significantly higher PWTs are noted in NP and TC animals as compared to UD animals at 8 weeks. NP, needle prick animals; NP+BUS, needle prick animals receiving buspirone; NP+OND, needle prick animals receiving ondansetron; PWT, paw withdrawal threshold; TC, tactile control animals; UD, undisturbed control animals. Data is presented as mean ± SEM, *P < 0.05.

During development from 3 to 8 weeks of age, PWT based on mechanical sensitivity to von Frey-filaments significantly increases over time for both paws [ipsilateral, F (3.048, 164.6) = 105.2, *p* < 0.01; contralateral, F (23.319, 179.2) = 81.69; *p* < 0.01]. The PWT increase over time seems dependent upon neonatal condition for the ipsilateral [interaction effect: F (20, 270) = 2.865, *p* < 0.01; Figure 4A] as well as the contralateral paw [interaction effect: F (20, 270) = 2.104, *p* < 0.01; Figure 4B]. NP+BUS animals show significantly lower ipsilateral PWTs compared to other groups at various time points: 3 weeks (NP+BUS 1.824 vs. NP 2.992; vs. NP+OND 3.411; vs. TC 3.189; vs. UD 3.576), 4 weeks (NP+BUS 3.103 vs. NP 3.626; vs. NP+OND 5.565; vs. TC 3.908; vs. UD 4.770) and 5 weeks (NP+BUS 3.876 vs. NP+OND 5.925). Contralateral PWTs of NP+BUS animals are significantly lower at 3 weeks (NP+BUS 1.787 vs. NP 3.103; vs. NP+OND 3.658, vs. UD 3.642) and 4 weeks (NP+BUS 2.876 vs. NP+OND 5.538, vs. UD 4.889). This effect ceases as NP+BUS animals grow older (>4–5 weeks of age). In addition, PWT of both hind-paws are significantly increased in NP and TC animals as compared to UD animals at 8 weeks of age (UD 6.682 vs. NP 9.622; vs. TC 10.90), suggesting differences in PWTs before incision. NP+OND animals do not show any differences in mechanical sensitivity from 3 to 8 weeks of age (*p* > 0.05).

### Duration of Mechanical Hypersensitivity to Re-injury in Adulthood After Repetitive Neonatal Needle Pricking Is Reduced by Neonatal 5-HT3R Antagonism, but Not 5-HT1aR Agonism

**Figure 5:**
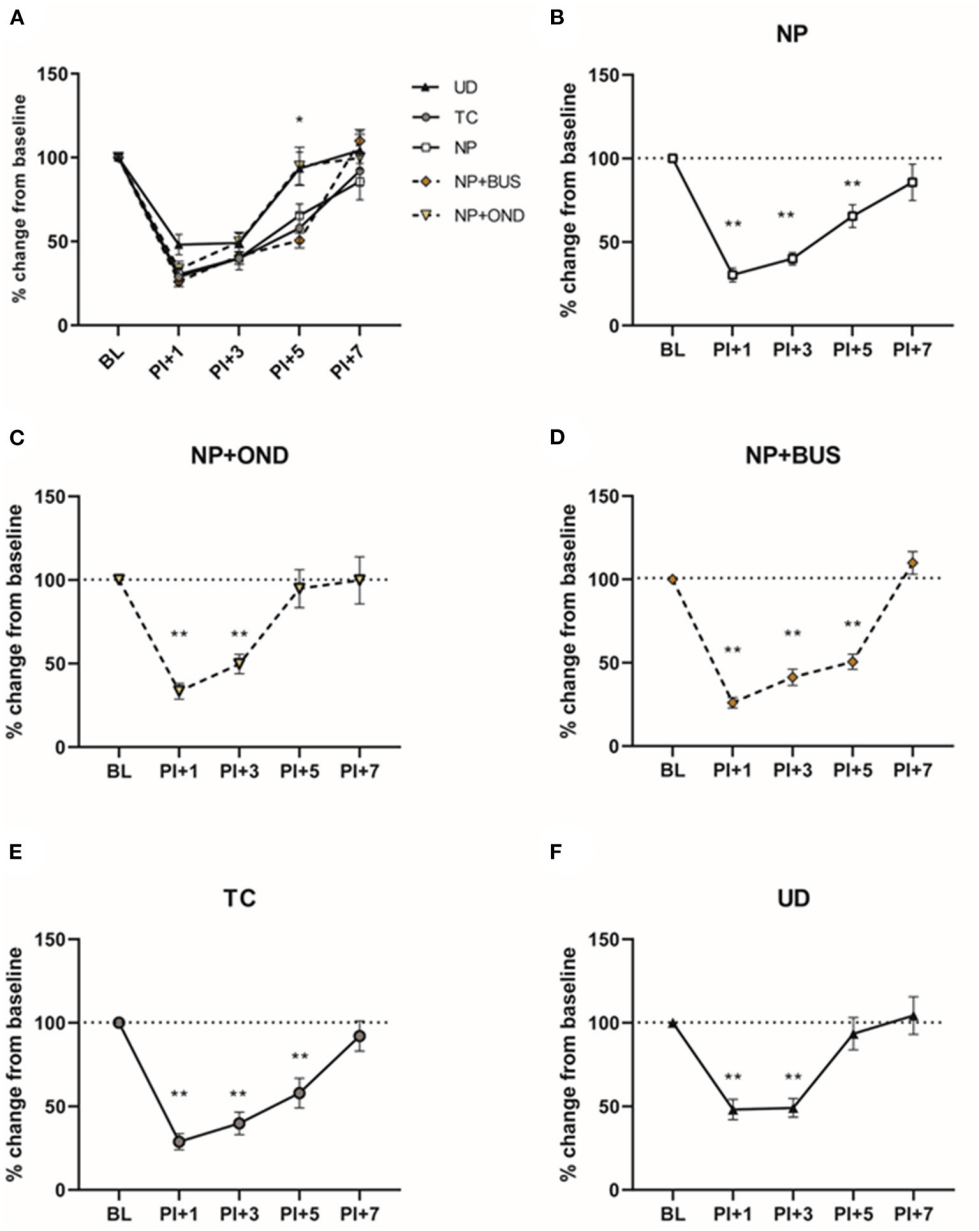
Mechanical sensitivity after re-injury in adulthood. **(A)** All groups show mechanical hypersensitivity 1 and 3 days after incision in adulthood, but differences emerge between groups at 5 and 7 days post-incision. Return to pre-operative base-line PWT levels is noted in UD animals by 5 days postoperative **(F)**, while PWTs of NP **(B)** and TC animals **(E)** are recovered to pre-operative baseline by 7 days postoperative. NP+OND **(D)**, but not NP+BUS **(C)**, abolishes this longer recovery time after incision. BL, baseline; NP, needle prick animals; NP+OND, needle prick animals receiving ondansetron; NP+BUS, needle prick animals receiving buspirone; PI, paw incision, +1, +3, +5; measurement 1, 3, and 5 days after paw incision; TC, tactile stimulation animals; UD, undisturbed control animals. Data is presented as mean ± SEM, *P < 0.05, **P < 0.01 as compared to their respective baseline.

The effect of re-injury to the same dermatome on the PWT is measured in adulthood from 1-day post-incision (PI+1) to 7 days post-incision (PI+7). All animals develop acute hypersensitivity 1 and 3 days after incision, however a significant interaction effect of time^*^condition is observed [F (16, 216) = 2.915; *p* < 0.01; Figure 5A]. Within group analysis shows that UD PWTs return to pre-incision values by 5 days post-incision (Figure 5F), whereas NP and TC animals return to baseline values by 7 days post-incision (Figures 5B,E). This increased duration of mechanical hypersensitivity after re-injury is abolished in NP+OND (Figure 5C), but not NP+BUS animals (Figure 5D), indicated by a recovery to pre-incision values at PI+5 rather than PI+7.

A significant interaction between time and condition is noted in contralateral PWTs [F (16, 216) = 1.891; *p* = 0.0225], with UD animals showing lower contralateral PWTs at PI+7 as compared to NP, NP+BUS and NP+OND animals. The injury-induced change from baseline PWTs is not significantly affected by condition and only shows a significant effect of time [F (2.877, 155.3) = 5.099; *p* = 0.0025, data not shown].

### Neonatal 5-HT1aR and 5-HT3R Modulation Affects Adult Anxiety Behavior in Males but Not Females

**Figure 6:**
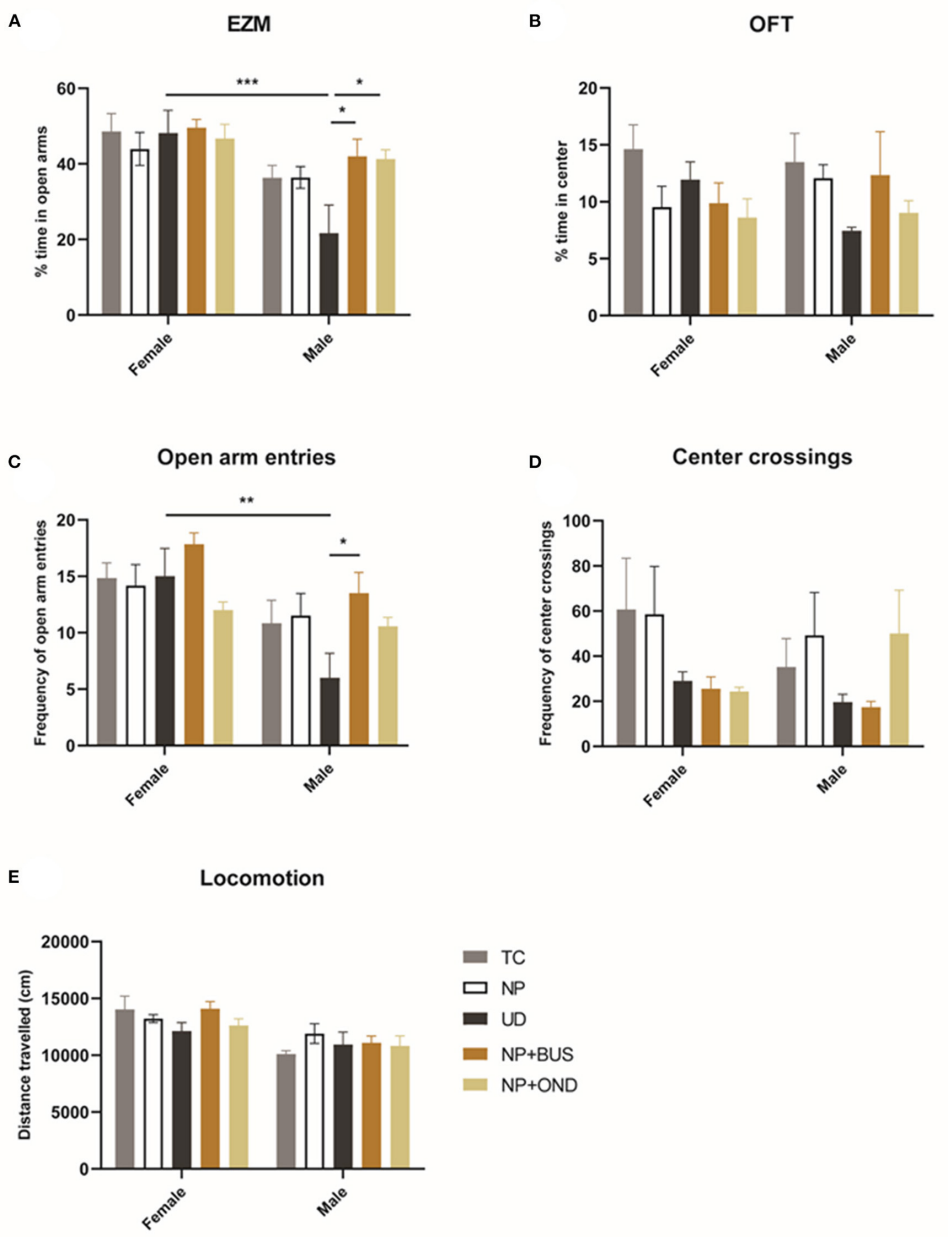
Neonatal conditions and adult anxiety behavior. **(A)** The percentage of time spend in the anxio-genic (open arm) region of the elevated zero maze (EZM) showed a significant difference between males and females [F (1, 49) = 19.75; *p* < 0.01]. No differences between conditions are observed in females, whereas UD males spend significant less time in the open arms as compared to all other neonatal conditions [F (4, 25) = 3.545; *p* = 0.0201]. **(B)** The percentage of time spend in the anxio-genic (center) region of the open field test (OFT) is not influenced by sex or condition. **(C)** The frequency of open arm entries is significantly affected by condition [F (4, 49) = 2.788; *p* = 0.0365] and sex [F (2, 49) = 16.62; *p* < 0.01]. UD animals exhibit significantly less open arm entries as compared to NP+BUS animals in males only. **(D)** The number of center crossings in the OFT is not affected by sex [F (1, 19) = 0.3304; *p* = 0.5681] or condition [F (4, 49) = 0.8757; *p* = 0.4853]. **(E)** Locomotor activity significantly differs between males and females [F (1, 49) = 21.20, *p* < 0.01] but is unaffected by condition [F (4, 49) = 1.177; *p* = 0.3325]. NP, needle prick animals; NP + OND, needle prick animals receiving ondansetron; NP+BUS, needle prick animals receiving buspirone; TC, tactile stimulation animals; UD, undisturbed control animals. Data is presented as mean ± SEM, *P < 0.05, **P < 0.01, ***P < 0.001.

In the OFT, no significant effect of condition [F (4, 49) = 1.972; *p* = 0.1135) or sex (F (1, 49) = 0.001; *p* = 0.9772; Figure 6B] on the percentage of time spend in the center of the arena is observed. In addition, the number of center crossings does not significantly differ between sex (F (1, 19) = 0.3304; *p* = 0.5681) or condition [F (4, 49) = 0.8757; *p* = 0.4853; Figure 6D]. Locomotor activity significantly differs between males and females [F (1, 49) = 21.20, *p* < 0.01] but is unaffected by neonatal condition [F (4, 49) = 1.177; *p* = 0.3325].

In the EZM, the percentage of time spend in the open arms of the EZM significantly differs between males and females [effect of sex: F (1, 49) = 19.75; *p* < 0.01]. Females do not show differences between conditions in the time spend in the open arms of the EZM (effect of condition: F (4, 24) = 0.2669; *p* = 0.8963). In males, UD spend significant less time in the open arms of the EZM as compared to NP+BUS and NP+OND animals [effect of condition: F (4, 25) = 3.545; *p* = 0.0201]. The number of open arm entries in the EZM reveals a significant effect of sex [F (2, 49) = 16.62; *p* < 0.01] and condition [F (4, 49) = 2.788; *p* = 0.0365; Figure 6C]. NP+BUS animals exhibit significantly more open arm entries as compared to UD animals, in males only (NP + BUS 13.50 vs. UC 6.00; *P* = 0.0266).

## Discussion

Neonatal repetitive needle pricking leads to acute hypersensitivity and increased duration of post-operative recovery after re-injury in adulthood (12–17, 23). Here, we show that selective neonatal agonism of the 5-HT1aR using buspirone reduces the acute hypersensitivity associated with neonatal procedural pain, whereas antagonizing the 5-HT3R using ondansetron attenuates the long-term consequences of neonatal procedural pain. Moreover, modulation of both neonatal 5-HT1aR and 5-HT3R is associated with decreased adult anxiety. Altogether, our data suggests that targeted pharmacological treatment directed at the serotonergic 5-HT1a and 5-HT3 receptors in neonates is possible and may be of use in treatment of acute neonatal procedural pain and its long-term consequences.

The 5-HT3R and 5-HT1aR are important in a variety of neurodevelopment processes (24, 46). This implies that early pharmacological interventions with buspirone or ondansetron may affect normal development of rat pups. For this reason, we controlled for major developmental milestones and possible side-effects in our study. Our results show that major developmental milestones, reflex development, weight and locomotor behavior were unaffected by early life repetitive procedures or 5HT3R and 5-HT1aR modulation, in line with earlier studies (47–51).

The observation that neonatal 5-HT1aR agonism using buspirone reverses acute hypersensitivity after repetitive needle pricking in rat pups can be explained by its inhibitory role in spinal neurotransmission, previously shown in neonatal dorsal horn cells (25). Here we show for the first time that this system can be used *in vivo* to attenuate sensitivity after noxious stimuli in neonatal animals, suggesting the inhibitory effect of the 5HT1aR is present and functional during the neonatal period. This is important as descending serotonergic RVM-spinal dorsal horn have been reported to act in a facilitatory way in neonates (26). In addition, the anti-nociceptive effect of 5-HT1aR becomes more apparent from postnatal day 4 onwards, and this coincides with progressive increase in acute hypersensitivity with cumulative exposure to repetitive needle pricking (14–16, 23). 5-HT1aR in neonates inhibit nociceptive signaling through a reduced glutamate release from primary afferent terminals or by increasing the incidence of long-term depression induced inhibition of postsynaptic dorsal horn responses (28, 29, 31, 32). 5-HT1aR mediated binding, via the use of selective agonists, thereby restores neonatal procedural pain-evoked disruptions in the normal balance of excitation and inhibition in the spinal dorsal horn (17, 33–35). 5-HT1aR is expressed on 70-80% of RVM neurons in neonatal rats (52), and may also mediate its anti-nociceptive effects by blocking descending serotonergic facilitation from the RVM observed in younger animals (25).

Our data indicates that acute hypersensitivity in rat pups exposed to neonatal procedural pain is likely not mediated via the 5-HT3R, as the neonatal 5-HT3R antagonist ondansetron showed no behavioral effect in our study. This is remarkable, as descending serotonergic release from the RVM are thought to play an important role in 5-HT3R mediated facilitation of nociceptive signaling up to P21 in rats (26, 53–55). 5-HT3R expression in the dorsal horn has been reported to be stable from P7 up to adulthood in rodents (26), but expression patterns are yet to be documented for the first postnatal week. Activation of neonatal 5-HT3R *in vitro* is involved in facilitation of most dorsal horn cells, although the level of facilitation remains modest (25, 28, 29). 5-HT3R function in the neonate is dose-dependent, facilitating nociceptive signaling at low levels while inhibiting at higher concentrations. This dual effect may be explained by an autoreceptor mediated function, where the 5-HT3R regulates basal release of 5-HT, GABA and glycine in the neonatal dorsal horn (30). Moreover, non-noxious mechanical stimuli, as used in this study, are not facilitated by descending serotonergic projections at P8 (26). In infant rats at P21, ondansetron reduces dorsal horn activity in response to nociceptive, non-nociceptive stimuli and mechanical stimuli (26). Thus it is likely, that although 5-HT3R antagonism may induce direct changes in the physiological response to nociceptive and non-nociceptive stimuli in neonates, this does not translate to a behavioral effect of 5-HT3R antagonism on mechanical hypersensitivity after neonatal procedural pain based on von Frey testing as in our model.

Neonatal procedural pain has been associated with changes in pain sensitivity that persist beyond infancy, in both clinical and preclinical settings (4–7). Here, we show that 5-HT3R antagonism during neonatal procedural pain prevents the increased duration of neonatal injury-induced postoperative hypersensitivity in adulthood (Figure 4). This suggests that the underlying injury-induced alteration in nociceptive pathways are regulated, at least in part, by neonatal 5-HT3R activation. The 5-HT3R plays a prominent role in network formation and function of the neonatal brain (46). Within the nociceptive network, 5-HT3R are located on primary afferent fibers, glutamate terminals and projection neurons in the dorsal horn (56–59). Neonatal injury increases the excitatory drive onto spinal sensory neurons, resulting in enhanced glutamate signaling (35, 60, 61), a process known to be of pivotal importance in sensitization of the nociceptive network (62). Descending serotonergic projections facilitate noxious spinal processing via the 5-HT3R in the dorsal horn in early life (26). Hence, the blocked facilitation of 5-HT3R during early neonatal procedural pain is likely to underlie the effect of ondansetron at later stages.

In contrast to the acute anti-nociceptive effect, neonatal 5-HT1aR agonism did not affect extended post-operative recovery in adulthood in our study. Chronic treatment with selective 5-HT1aR agonists for 7 days, like in our study, desensitizes 5-HT1a auto-receptors, leading to impaired functioning of this receptor (63–65). Likewise, substance P that is released upon nociceptive C-fiber activation, likely to occur after repetitive needle pricking, also desensitizes 5-HT1aR and may impact receptor functioning (66, 67). Future studies should include anatomical as well as functional analysis to detect changes in 5-HT1aR functioning after neonatal targeting. During development, we observed a temporary increase in mechanical sensitivity in NP animals when exposed to neonatal 5-HT1aR agonist buspirone that ceased with increasing age (>5 weeks). Repetitive activation of the 5-HT1aR from P0 to P7 may result in long-term depression (28), leading to altered synaptic plasticity in the spinal nociceptive circuit which consequently may alter basal nociceptive behavior.

Serotonin signaling also regulates the normal development of emotional behavior, including anxiety (24). Hence, sensitive windows in development through which serotonergic signaling is altered may differentially encode anxiety behaviors. Neonatal 5-HT1aR agonism decreased adult male anxiety in the EZM as compared to undisturbed males, but did not affect anxiety-related behavior in the OFT (Figure 6). 5-HT1aR have been reported to be central players in controlling anxiety (36), and thus interference with early postnatal functioning of the 5-HT1aR (buspirone) may affect normal anxiety behavior (36, 37). Neonatal 5-HT3R antagonism using ondansetron also shows some anxiogenic properties in males in the EZM, albeit small. Previous studies on the role of 5-HT3R antagonism related to anxiety remain contradictory (38, 68), Of note, our results demonstrate the absence of any difference in adult state and trait anxiety levels between TC and NP animals and neonatal 5-HT1aR or 5-HT3R modulation, suggesting receptor modulation does have an additive effect on anxiety. Interestingly as related to anxiety-related behavior as measured using the EZM, a significant difference between sex in anxiety is noted that was not observed or assessed in earlier studies using a similar neonatal model (20, 22, 23, 50). Lower levels of anxiety in females compared to males, predominantly observed in the undisturbed animals in our study, is not uncommon in rodent models that use both sexes (69).

The current study is not without limitations. First, the specificity of buspirone and ondansetron as pharmacological modulators of the 5-HT1aR and 5-HT3R, respectively, is of importance. Buspirone has high affinity for 5-HT1aR but also increases dopamine levels via D2 inactivation as well as noradrenaline levels via α2R in the frontal cortex (70, 71). Therefore, buspirone-mediated effects in our study may not be uniquely related to 5-HT1aR functioning, but can also be (partially) the result of D2R or α2R binding. Ondansetron is a potent, highly selective antagonist of the 5-HT3R (72). Although it shows some affinity for 5-HT1b, 5-HT4, opioid and α1-adrenergic receptors, it has a selectivity of 1,000:1 toward 5-HT3R (40). Hence, the effects of ondansetron as observed in this study are most likely exclusively mediated by 5-HT3R binding. Next, ondansetron and buspirone were administered systemically and this limits the interpretation of location-specific effects in the spinal cord nociceptive network. Future studies should include local intrathecal administration to further specify the involvement of spinal 5-HT1aR and 5-HT3R in the observed effects. Lastly, baseline differences between animals exposed to repetitive needle pricking or tactile stimulation and undisturbed animals at 8 weeks of age, prior to re-incision were observed Nevertheless and based on within-group analysis any possible effects related to the differences in baseline value differences on post-operative mechanical sensitivity can be excluded (14–16).

## Conclusion

Our data shows that neonatal targeted pharmacological modulation of the 5-HT1aR and 5-HT3R attenuates the acute and long-term effects associated with neonatal procedural pain. These results may form the fundament of a new mechanism-based therapeutic venue in treatment of procedural pain in human neonates.

## Data Availability Statement

The raw data supporting the conclusions of this article will be made available by the authors, without undue reservation.

## Ethics Statement

The animal study was reviewed and approved by Committee for Experiments on Animals, Maastricht University, Maastricht, Netherlands (DEC 2017-017).

## Author Contributions

AK, EJ, JP, DT, and NH conceived the research. AK, EJ, and NH designed and conceptualized the research and contributed to its structure and content. AK performed the research, analyzed the data, and drafted the manuscript and figures. EJ provided the resources for this research. All authors commented on previous versions of the manuscript, critically revised, and quality assessed the manuscript. All authors have read and approved the final version of the manuscript.

## Funding

AK received financial support for this research provided by the Pain Knowledge Centre from Maastricht and an institutional grant from University Maastricht, School of Mental Health and Neuroscience.

## Conflict of Interest

The authors declare that the research was conducted in the absence of any commercial or financial relationships that could be construed as a potential conflict of interest.

## Publisher's Note

All claims expressed in this article are solely those of the authors and do not necessarily represent those of their affiliated organizations, or those of the publisher, the editors and the reviewers. Any product that may be evaluated in this article, or claim that may be made by its manufacturer, is not guaranteed or endorsed by the publisher.
